# Gossypiboma Complicating as Colouterine Fistula in a Young Woman Post-Cesarean Section

**DOI:** 10.7759/cureus.17846

**Published:** 2021-09-09

**Authors:** Pratik K Jha, Awgesh Verma, Mumtaz A Ansari, Vivek Srivastava

**Affiliations:** 1 Department of General Surgery, Institute of Medical Sciences, Banaras Hindu University, Varanasi, IND

**Keywords:** gossypiboma, obstruction, perforation, colo-uterine fistula, diverticulitis, lower segment caesarean section

## Abstract

Gossypiboma is a mass of foreign body with cotton matrix accidentally left inside the body after a surgical procedure. It is a surgeon’s nightmare and has a varied presentation ranging from asymptomatic cases to the formation of an abscess, mass, intestinal obstruction/perforation, malabsorption, gastrointestinal hemorrhage, and various internal and external fistulization. Genital tract fistulas are one of the most distressing conditions for women of reproductive age that not only hamper their day-to-day work but also impair their social life and psychological state. Colouterine fistula is a rare pathology and has been mainly reported as a complication of diverticulitis in the elderly. We present here a case of gossypiboma presenting as colouterine fistula in a young lady following lower segment cesarean section. The case highlights a rare complication of gossypiboma, probably the first of its kind, and the diagnostic challenges that it presents.

## Introduction

Gossypiboma (textiloma, gauzeoma, cottonoid) is described as a mass of a foreign body with a cotton matrix left inside the body cavity during a surgical procedure. The reported incidence of this misevent is between one and 1000-1500 abdominal surgeries [1]. It may remain asymptomatic or present from months to years after incident surgery in the form of an abscess, mass, intestinal obstruction/perforation, malabsorption, gastrointestinal hemorrhage, and fistula formation [1]. Among various internal fistulizations, colouterine fistula is rare due to the thick muscular wall of the uterus that provides a protective barrier [2]. It has most commonly been reported as a complication of diverticulitis in the elderly with other rare causes being sigmoid malignancy, radiotherapy, iatrogenic (intrauterine device, endometrial curettage), and obstetrical injury [2].

## Case presentation

A 28-year-old woman presented with complaints of pain and progressively increasing swelling in the left side of the lower abdomen for three months. She also complained of fecal discharge with the periodic involuntary escape of gas through the introitus. She had undergone emergency lower segment cesarean section for obstructed labor four months back with intraoperative hemorrhage managed successfully with blood transfusion and pressure application.

Abdominal examination revealed a firm lump of size 15 x 10 cm in the left lower abdomen with the lower border going into the pelvis. Per-speculum and per-vaginal examination showed the vaginal cavity filled with pus-mixed fecal content with a bimanually palpable mass in the left fornix.

**Figure 1:**
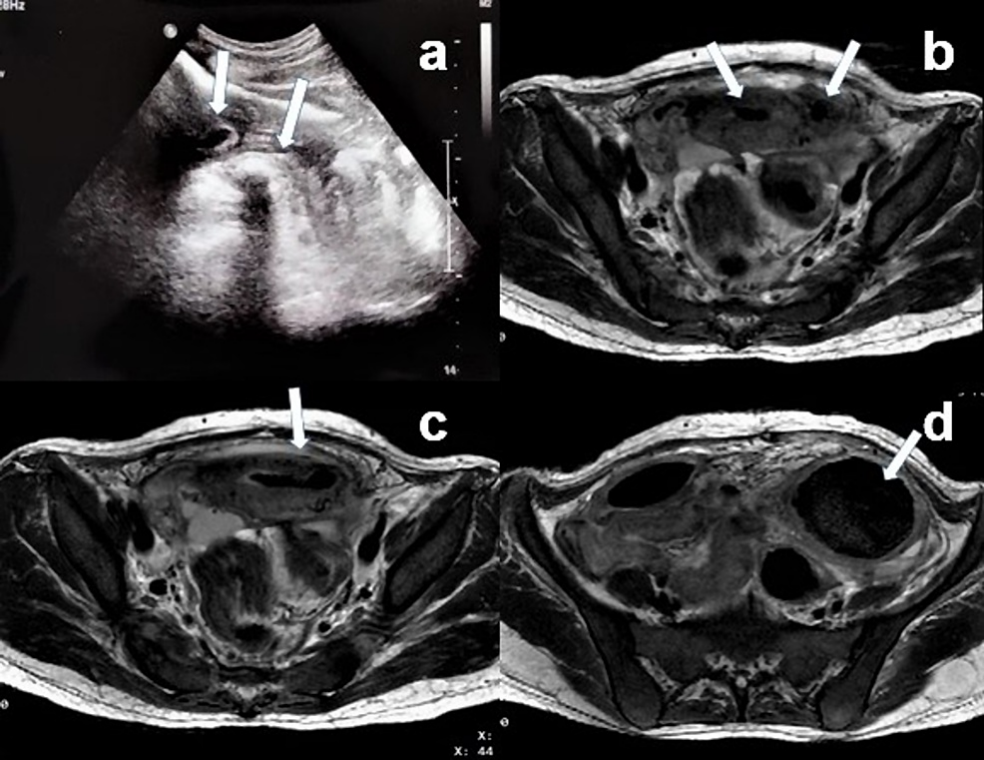
Ultrasonography and magnetic resonance imaging with fistulography (a) Ultrasound of abdomen showing a bulky uterus with intraluminal air foci (left arrow) and a hyperechoic mass with posterior acoustic shadowing in left parauterine space (right arrow). (b) MRI lower abdomen with fistulogram showing intraluminal air foci in the uterus, and (c) a fistulous tract between endometrial cavity (left arrow) and adjacent sigmoid colon (right arrow) in the left cornu region. (d) A mass with whorled stripes (arrow) in a fluid-filled cavity with low signal in the peripheral wall on axial TSE-T2 weighted image suggestive of gossypiboma.

Ultrasonography (USG) of the pelvis revealed air foci in the endometrial cavity with multiple surrounding loculated abscesses and a hyperechoic mass with posterior acoustic shadowing in the left parauterine space (Figure 1A). Magnetic resonance imaging (MRI) revealed a fistulous tract connecting the left cornu of the uterus with the adjacent sigmoid colon on T2-weighted short-tau inversion recovery (T2w-STIR) imaging (Figures 1B-1C) and a mass with whorled stripes in a fluid-filled cavity with low signal in the peripheral wall on axial T2-weighted turbo spin-echo (TSE) imaging suggestive of a foreign body (Figure 1D).

**Figure 2:**
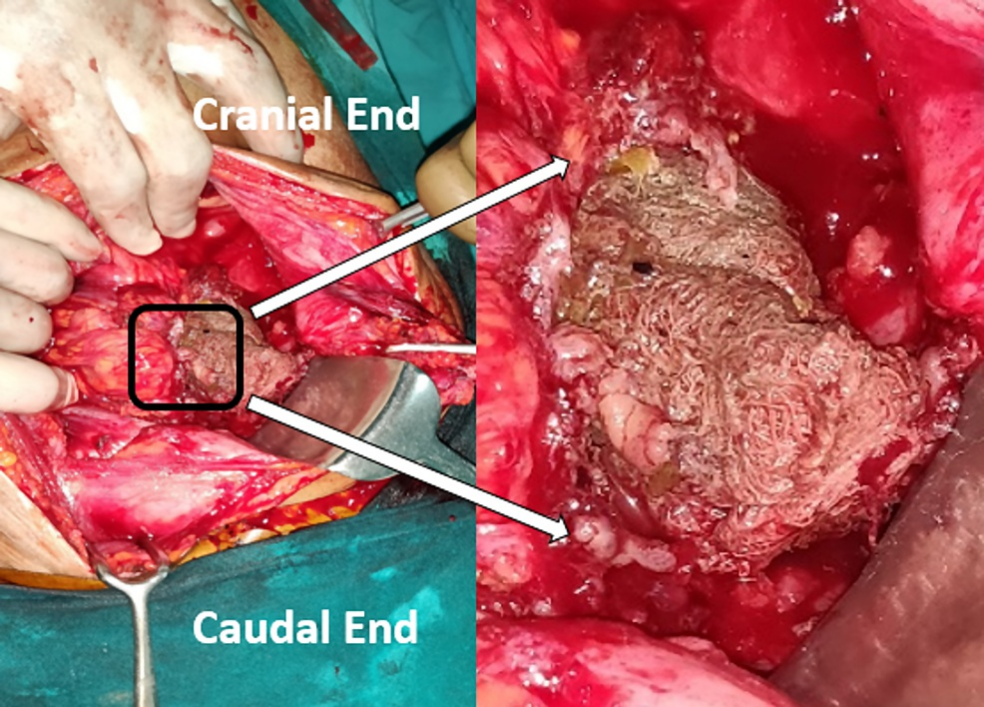
Intraoperative finding Encountered surgical sponge after exploration and meticulous adhesiolysis

**Figure 3:**
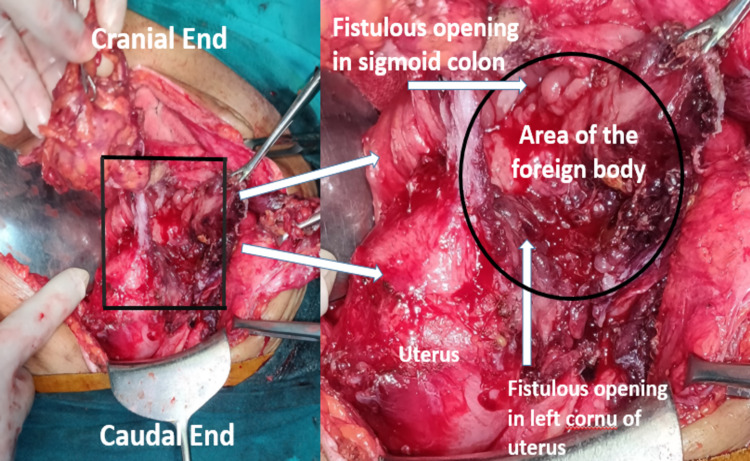
Intraoperative anatomy after removal of gossypiboma Site of gossypiboma with colo-uterine fistula. Note the adjacent openings on the medial aspect of the adhered sigmoid colon and the left cornu of the uterus.

**Figure 4:**
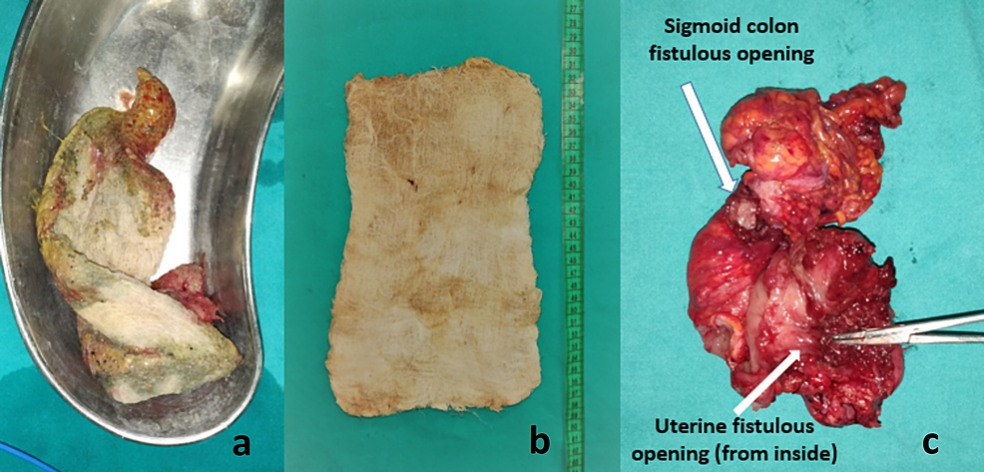
Retrieved foreign body and gross pathological specimen (a) Retrieved specimen of retained surgical sponge (gossypiboma), (b) without radiopaque thread, (c) gross specimen of resected colouterine fistula

The patient was planned for exploration with consent for stoma and hysterectomy. Laparotomy revealed a thick-walled abscess cavity in the lower abdomen surrounding a surgical sponge in the left parauterine space (Figure 2). It had eroded the anterior sigmoid colonic wall and the left cornu of the uterus (Figure 3). It was removed after adhesiolysis followed by resection of colouterine fistula (Figure 4) with end sigmoid colostomy due to unhealthy bowel and peritonitis. The postoperative course was uneventful. The patient has been doing well at two months of follow-up and is waiting for colostomy takedown.

## Discussion

Gossypiboma often becomes a differential diagnosis, by exclusion, of soft tissue masses or localized abdominal pain in a patient with a history of prior operation. The well-known risk factors are emergency surgery, change in surgical team, obesity, hemorrhage, and a high count of sponge/instruments used [3]. In our patient, the apparent risk factor was the emergency indication of the cesarean section and the intraoperative hemorrhage.

Clinical presentation depends upon the location of the foreign body and the type of inflammatory response. Gauze and cotton pads can cause aseptic fibrous or exudative responses [4]. The fibrous type presents with adhesions, encapsulation, and eventually granuloma formation, whereas the exudative type occurs early in the postoperative period, resulting in abscess formation and may involve secondary bacterial infection. This results in the formation of various fistulas seen in gossypiboma [4]. In our case, it may be inferred that the aseptic fibrotic response led to the formation of a granulomatous mass; continued inflammation caused adhesion of sponge material to the adjacent sigmoid colon and the uterus, which could have gradually eroded the adjoining walls creating a colo-uterine fistula with superimposed infection.

Diagnosis of gossypiboma is not straightforward because the cotton can simulate hematoma, granulomatous process, abscess formation, cystic masses, or neoplasm [5]. Although X-ray, USG, computed tomography (CT), MRI, colonoscopy, hysteroscopy, and others aid in the diagnosis, they are often non-specific. On plain X-ray, gossypiboma may be identified as curved or banded radio-opaque lines if it has a radiological marker. The USG feature is usually a well-defined mass containing wavy internal echogenic focus with a hypoechoic rim and a strong posterior acoustic shadow [6]. On CT, it may manifest as a cystic lesion with an internal spongiform appearance with mottled shadows as bubbles mimicking a fecaloma, hyperdense capsule, and concentric layering [7]. MRI is a versatile, detailed, and accurate diagnostic tool in diagnosing a retained foreign object as well as a colouterine fistula. MRI features of gossypiboma include a well-defined mass with a peripheral wall of low signal intensity on T1- and T2-weighted imaging, with whorled stripes seen in the central portion and peripheral wall enhancement after intravenous gadolinium administration on T1-weighted imaging [8]. In our patient, MRI helped in establishing the diagnosis.

Among the various treatment options for colouterine fistula, Hartman’s procedure, transverse colostomy, closure of the fistula without bowel resection, and en bloc resection of the uterus and sigmoid colon are several available surgical treatment options [3,7-8]. Although en bloc resection or adding hysterectomy may be justified in malignancy, in benign conditions, the need for a hysterectomy has not been established. However, the patient should be counseled in the perioperative period regarding the high risk of infertility although one may still be able to conceive. In the present case, retrieval of the foreign object with excision of the fistulous segment and end colostomy was done in the best interest of the patient.

## Conclusions

Gossypiboma should be included in the differential diagnosis of soft tissue masses or localized abdominal pain in a patient with a history of prior operation. The diagnosis is often difficult to make. Fecal discharge per vaginum can be a presentation of a rare pathology like colouterine fistula. Counting gauges, sponges, and instruments before and after the surgery; inspection of the operative field thoroughly before closing; using radio-opaque markers and X-rays of the abdomen before and after fascial closure; avoiding a change of surgical team/scrub nurse in between the procedure; avoiding communication errors and implementing the WHO Surgical Safety Check List in all surgical procedures are some of the proven methods for prevention of a retained foreign body.
